# Meta-Analysis of Bioaccessibility of Hydrophobic Compounds in Buttermilk Matrices: A Systematic Review and Quantitative Synthesis

**DOI:** 10.3390/molecules31091526

**Published:** 2026-05-04

**Authors:** Assem Sagandyk, Tamara Tultabayeva, Gulmira Zhakupova, Kadyrzhan Makangali, Aknur Muldasheva, Aruzhan Shoman, Kalamkas Dairova

**Affiliations:** 1 Department of Food Production, Saken Seifullin Kazakh Agrotechnical University, Astana 010000, Kazakhstan; 2 International School, University of California, Davis, 1 Shields Ave, Davis, CA 95616, USA; 3 Scientific and Innovation Center Agro Tech, Astana IT University, Astana 010000, Kazakhstan

**Keywords:** bioaccessibility of buttermilk, milk fat globule membrane (MFGM), hydrophobic compounds, meta-analysis

## Abstract

Hydrophobic bioactive compounds, such as curcuminoids, β-carotene and long-chain lipids, as well as amphiphilic structural lipids (milk fat globule membrane (MFGM)-associated phospholipids), often exhibit low bioaccessibility due to poor aqueous solubility and/or susceptibility to degradation, which limits their effective use in functional foods. Buttermilk, a dairy byproduct enriched with proteins, lipids and MFGM components, provides a structurally complex, amphiphilic matrix that can enhance micellar solubilization, protect hydrophobic and amphiphilic compounds during digestion and thereby modulate their potential bioavailability. This systematic review and meta-analysis, conducted and reported in accordance with the PRISMA 2020 guidelines, synthesizes quantitative data from in vitro gastrointestinal digestion studies to evaluate the impact of buttermilk and related matrices (e.g., buttermilk yogurt, ultrafiltered buttermilk, and composite nanosystems) on the bioaccessibility of hydrophobic compounds and MFGM phospholipids compared with aqueous or non-buttermilk controls. We identified a limited but growing body of in vitro evidence indicating that buttermilk-based matrices generally increase the intestinal bioaccessibility of curcuminoids, β-carotene, omega-3 fatty acids, vitamin and MFGM phospholipids relative to non-buttermilk systems, with particularly pronounced effects in structured emulsions, yogurts, ultrafiltered buttermilk and MFGM-enriched nanosystems. Rather than a single effect size, the data point to a compound- and matrix-dependent spectrum of improvements, influenced by both the chemical nature of the bioactive and the supramolecular organization of the dairy matrix. Mechanistically, the available findings support a plausible hypothesis that buttermilk enhances bioaccessibility via MFGM-mediated micellar solubilization, interfacial protection against pH- and enzyme-driven degradation and favorable lipid partitioning, although these pathways remain to be confirmed in dedicated mechanistic and in vivo studies. Methodological heterogeneity and the exclusive reliance on in vitro models are important limitations, but overall, the synthesis supports buttermilk and MFGM-rich ingredients as sustainable, food-grade carriers for lipophilic nutraceuticals and highlights the importance of dairy matrix structure in the design of functional delivery systems.

## 1. Introduction

Buttermilk, a versatile dairy byproduct, has garnered significant attention in food science and nutrition due to its unique composition and potential health benefits. Defined as the aqueous phase separated during the churning of cream in butter production, buttermilk arises from the disruption of milk fat globules, releasing membrane components into the liquid fraction [1,2]. This process distinguishes it from fermented or cultured buttermilk, which may involve acidification but shares similar foundational characteristics. Historically, buttermilk was a staple in traditional diets, valued for its tangy flavor and digestibility, but modern industrial practices have repositioned it as a valuable resource in functional foods and nutraceuticals [3].

The production of buttermilk primarily occurs as a secondary output in dairy processing. In butter manufacturing, cream—typically containing 30–40% fat—is churned mechanically, causing the fat globules to aggregate and separate from the aqueous phase. This yields sweet buttermilk from uncultured cream or sour buttermilk from cultured variants, where lactic acid bacteria are added to lower the pH and enhance flavor. Globally, approximately 6.5–7.0% of milk production is dedicated to butter, generating around 3.2 million tons of buttermilk annually [4]. In the European Union alone, production reached 0.5 million tons in 2016 [5]. Variations include whey buttermilk, derived from whey cream during cheese production, which exhibits higher phospholipid and lactose content but lower nitrogen levels compared to sweet or sour types. Industrial valorization often involves concentration techniques such as microfiltration (MF), ultrafiltration (UF), or diafiltration (DF) to enrich bioactive fractions [6]. For instance, UF can concentrate buttermilk two-fold, improving its coagulation properties for applications like cheese making while maintaining salt balance and minimizing waste. Pretreatments such as acid precipitation or citrate addition remove caseins, optimizing phospholipid extraction via UF, while supercritical fluid extraction further refines polar lipids, highlighting buttermilk’s role in sustainable dairy waste management [7].

In this context, bioaccessibility is defined as the fraction of an ingested compound that is released from the food matrix into the gastrointestinal fluid and becomes available for intestinal absorption, typically operationalized as the proportion solubilized in the intestinal mixed micellar phase after in vitro digestion. Although bioaccessibility does not guarantee systemic bioavailability, it represents a critical prerequisite step and a widely used proxy endpoint in in vitro digestion models [8].

Compositionally, buttermilk mirrors skim milk in many aspects but is distinguished by the presence of the milk fat globule membrane (MFGM), which constitutes a tri-layer structure encapsulating fat globules in raw milk [9]. Upon churning, MFGM fragments (0.1–3.0 μm) disperse into buttermilk, enriching it with polar lipids; within the lipid fraction, approximately 26–31% are phospholipids (including phosphatidylethanolamine, phosphatidylcholine, sphingomyelin, phosphatidylinositol and phosphatidylserine), while about 62% and 9% correspond to tri- and diglycerides, respectively. On a dry matter basis, buttermilk typically contains 31.5–33.1% protein, 48.7–53.8% lactose, 5.7–13.1% fat, minerals and minor components such as vitamins, enzymes and bioactive peptides [10]. Milk fat globule membrane-associated phospholipids are substantially enriched in buttermilk compared with the original milk, reflecting their preferential partitioning into the serum phase during churning [5]. Proteins include whey components like β-lactoglobulin and α-lactalbumin, alongside MFGM-specific glycoproteins such as butyrophilin (BTN), xanthine oxidase/dehydrogenase (XO/XDH), mucins (MUC1 and MUC15) and adipophilin (ADPH) [4,10]. Minor elements like vitamins (e.g., riboflavin), enzymes, and bioactive peptides further enhance their profile. In dry form, polar lipids range from 1.2 to 2.1%, and the overall dry matter fat content varies from 4.6 to 14.5%. Whey buttermilk differs by having elevated phospholipids and lactose but reduced nitrogen, influenced by the source material [3].

Beyond its bulk composition, buttermilk is characterized by a complex interfacial architecture derived from milk fat globule membrane (MFGM) fragments, which are rich in phospholipids, glycolipids and glycoproteins. These amphiphilic molecules assemble at oil–water interfaces and within colloidal structures, thereby governing droplet stability, interfacial curvature, and the interactions of buttermilk emulsions with digestive surfactants and enzymes [9,10].

Applications of buttermilk span food, pharmaceutical, and industrial sectors, leveraging its emulsifying, stabilizing, and bioactive properties [11]. In the food industry, it serves as a base for beverages, including carbonated fruit-flavored drinks (e.g., optimized with 24% pineapple juice and 12% sugar), probiotic-enriched fermented products (e.g., with Aloe vera or moringa for extended shelf-life), and fiber-fortified variants to reduce phase separation. This ingredient enhances baked goods by increasing water absorption, protein content, and rheological properties like dough development time and gelatinization temperature; at 30% incorporation, it improves texture, volume, and sensory attributes in bread, flatbread, and chocolate. As a microencapsulation matrix, its protein- and MFGM-rich walls encapsulate hydrophobic compounds like omega-3 oils, curcuminoids, trans-resveratrol, and probiotics (e.g., *Lactobacillus rhamnosus* GG), achieving high efficiency (up to 94.61%) and stability via spray drying or homogenization [4,7]. It supports microbial fermentation for exopolysaccharides, organic acids, and essential fatty acids like DHA [12]. Industrially, buttermilk forms biofilms with corn starch for sustainable packaging, exhibiting Newtonian rheology and antioxidant activity without antimicrobial effects against *Listeria innocua*. It inhibits bacterial adhesion on stainless-steel surfaces for up to 720 min, outperforming skim milk. In pharmaceuticals, MFGM extracts are incorporated into infant formulas (e.g., Lacprodan^®^ MFGM-10) to promote cognitive development and reduce diarrhea [13]. Cosmetics and oral care utilize phospholipid extracts, while edible films from whey–buttermilk blends offer barrier properties. These uses address dairy waste valorization, reducing environmental pollution and economic losses while creating value-added products [3,4].

In this context, buttermilk-derived matrices provide a unique combination of phospholipids, proteins and finely dispersed fat droplets that can promote micellar solubilization, protect hydrophobic compounds from degradation and alter their partitioning between oil, aqueous and micellar phases during digestion. Hydrophobic compounds, including curcuminoids (e.g., curcumin), carotenoids (e.g., β-carotene), omega-3 fatty acids and vitamins such as D_3_, possess valuable health benefits such as antioxidant, anti-inflammatory and anticancer properties; however, their low water solubility and rapid degradation in aqueous environments result in poor bioaccessibility and subsequent bioavailability. MFGM-rich dairy matrices such as buttermilk, rich in amphiphilic components (e.g., phospholipids, proteins and MFGM), can facilitate micellar solubilization, protect against degradation and enhance intestinal absorption [14]. In this work, we therefore use the broader term “lipophilic bioactives” to encompass both strictly hydrophobic compounds (curcuminoids, β-carotene, long-chain omega-3 fatty acids, and vitamin D_3_) and amphiphilic structural lipids (MFGM-associated phospholipids), which all share a common dependence on lipid digestion and mixed micelle formation for intestinal bioaccessibility in buttermilk matrices.

This systematic review and meta-analysis quantify the bioaccessibility of hydrophobic compounds in buttermilk-based systems using in vitro models, addressing gaps in understanding their delivery potential in functional foods such as yogurts, cheeses and nanosystems [15,16].

## 2. Materials and Methods

### 2.1. Search Strategy

A systematic literature search was conducted in accordance with the Preferred Reporting Items for Systematic Reviews and Meta-Analyses (PRISMA) guidelines to identify relevant studies on the bioaccessibility of lipophilic compounds in buttermilk or buttermilk-derived matrices. The primary literature search was performed in PubMed and Scopus, complemented by a manual screening of reference lists and targeted searches in Google Scholar to capture additional or recently published records. The search was limited to articles published in English from 2010 to 2025 to capture recent advancements in food matrix research. Reference lists of included studies and relevant reviews were manually screened for additional eligible publications. Narrative and systematic review articles were excluded from the quantitative synthesis and were used only to contextualize and interpret the findings of primary in vitro studies. For PubMed and Scopus, we used combinations of terms related to the matrix and outcome, for example, (“buttermilk” OR “buttermilk powder” OR “milk fat globule membrane” OR “MFGM”) AND (“bioaccessibility”) AND (“in vitro digestion” OR “simulated digestion”), which were further combined with specific compound terms (e.g., “curcumin” OR “curcuminoids” OR “β-carotene” OR “omega-3” OR “vitamin D_3_” OR “phospholipids”). The review was not prospectively registered in a public database.

### 2.2. Inclusion and Exclusion Criteria

Inclusion criteria required studies to: (1) Report quantitative in vitro digestion data on bioaccessibility (expressed as percentages) of hydrophobic compounds (e.g., curcuminoids, β-carotene, long-chain omega-3 fatty acids, and vitamin D_3_) in buttermilk or derived matrices (e.g., yogurt, nanosystems, and ultrafiltered products). For the purposes of this review, amphiphilic structural lipids (MFGM phospholipids) were also included under a common lipophilic category at the screening stage but were treated as distinct subgroups in all quantitative analyses, with phospholipids pooled and interpreted separately from neutral hydrophobics. (2) Include comparisons to controls (e.g., aqueous dispersions or non-buttermilk matrices). (3) Use standardized in vitro models, such as the INFOGEST protocol for static gastrointestinal simulation [17].

For subgroup analyses, studies were further stratified by target compound (curcuminoids, β-carotene, omega-3 fatty acids, vitamin D_3_, and MFGM phospholipids) and buttermilk matrix type (plain buttermilk, yogurt/emulsions, nanosystems, and UF/MFGM-enriched), with the “curcuminoid subgroup” defined as studies meeting the above criteria specifically for curcuminoids in buttermilk or buttermilk–yogurt matrices and reporting intestinal mixed micellar bioaccessibility suitable for pooling.

Exclusion criteria encompassed: (1) in vivo-only studies; (2) non-dairy matrices without buttermilk components; (3) qualitative reports without numerical data; and (4) studies not focused on hydrophobic bioactives. Study selection was performed independently by two reviewers, with discrepancies resolved through discussion.

### 2.3. Study Quality and Risk of Bias Assessment

Study quality and risk of bias were appraised qualitatively based on the reporting of digestion conditions (e.g., bile concentration and fed/fasted state), analytical methods, and completeness of outcome data; however, no formal scoring system was applied, given the small number and methodological diversity of in vitro studies.

### 2.4. Data Extraction

Data were extracted independently by two reviewers using a standardized form. Extracted variables included: study characteristics (year, compound, matrix, digestion model, conditions such as fasted/fed states, bile extract levels [0–40 mg/mL], and ethanol presence [0–2% *v*/*v*]); intestinal bioaccessibility percentages (means ± SD or ranges), defined as the percentage of the initial dose recovered in the small-intestinal mixed micellar fraction at the end of the simulated intestinal phase; degradation/stability metrics (e.g., percentage loss post-digestion); and sample sizes (replicates) [18,19]. Data were grouped by compound type (e.g., curcuminoids, β-carotene) and matrix.

### 2.5. Statistical Analysis

Due to anticipated heterogeneity from varying models and conditions, a random-effects meta-analysis was applied using inverse-variance weighting [20]. Pooled means and standard errors (SEs) were calculated as follows:

(1) $$\bar{x}=\frac{\sum \left(x_{i}*w_{i}\right)}{\sum w_{i}},\;\;w_{i}=\frac{1}{\sigma_{i}^{2}},\;\;\sigma_{p}^{2}=\frac{1}{\sum w_{i}}$$

where x_i_ is the mean bioaccessibility, σi is SD, and the DerSimonian–Laird estimator was used for between-study variance (τ^2^) [21].

Between-study variance (τ^2^) was estimated using the DerSimonian–Laird method and incorporated into the random-effects weights. Specifically, τ^2^ was calculated as follows:

(2) $$\tau^{2}=\max\left\{0,\frac{Q-(k-1)}{\sum w_{i}-\frac{\sum w_{i}^{2}}{\sum w_{i}}}\right\}$$

where Q is Cochran’s Q statistic, *k* is the number of studies, and w_i_ are the fixed-effect weights.

Random-effects weights were then obtained as w_i_ = 1/(σ_i_^2^ + τ^2^).

Heterogeneity was assessed using I^2^ (high if >75%) and Q statistics. Subgroup analyses were performed by compound and conditions (e.g., fasted/fed). Sensitivity analyses excluded studies with approximated SDs to test robustness. Meta-analyses were implemented in Python (v.3.11) using the numpy and scipy libraries, following standard random-effects procedures, with all calculations performed via custom scripts that are available from the authors upon request.

In this context, “pooled mean bioaccessibility” refers to the random-effects weighted mean of study-specific bioaccessibility values, where each study’s mean (and corresponding variance) contributes to an overall estimate that summarizes the central tendency across eligible studies, together with its 95% confidence interval.

### 2.6. Comparative Analysis Methods

To benchmark buttermilk against other delivery systems, a parallel search was conducted using modified terms (e.g., “bioaccessibility curcuminoids in vitro”). Categories included oil-based emulsions, protein-based matrices (e.g., casein and whey), phospholipid systems (e.g., soy lecithin liposomes), polysaccharide complexes, and synthetic nanoparticles (e.g., SLN/NLC). Inclusion mirrored the primary criteria, with pooled estimates for each category using the same inverse-variance method [22]. Indirect comparisons focused on overlapping confidence intervals and fold increases vs. controls, prioritizing head-to-head data where available [8].

### 2.7. Degradation and Stability Analysis Methods

Degradation was quantified as the percentage loss of the parent compound across digestion phases, with stability as retention rates post-digestion. Data were pooled using random-effects modeling [20]. Heterogeneity was evaluated with I^2^. Phase-specific analyses (oral, gastric, intestinal, and colonic) incorporated fecal fermentation where reported, following INFOGEST extensions [17]. Pooled metrics were derived for curcuminoids and β-carotene, with comparisons to non-matrix controls.

In practice, the intestinal mixed micellar fraction at the end of simulated gastric–intestinal digestion was the most consistently reported final bioaccessibility endpoint across studies, and we therefore used this phase as the common reference point for quantitative pooling and comparison between compounds and carrier systems. Accordingly, only intestinal mixed micellar bioaccessibility values (end of gastric–intestinal digestion) were pooled using the random-effects model, whereas phase-specific values from earlier or colonic stages were not pooled quantitatively but were used solely for qualitative interpretation.

## 3. Results

### 3.1. Study Selection and Characteristics

The primary meta-analysis was restricted to buttermilk and buttermilk-derived matrices; other dairy or non-dairy systems were only used for qualitative and indirect quantitative comparison.

**Figure 1:**
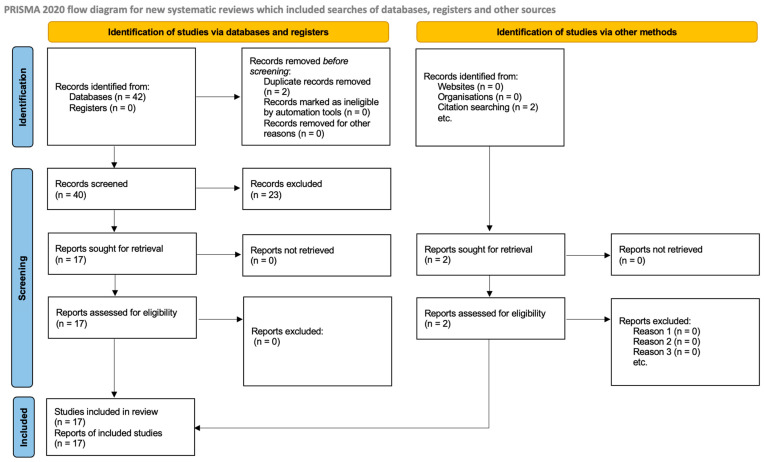
PRISMA 2020 flow diagram of the study selection process.

A total of 42 records were identified through database searches, including an expanded search targeting recent advancements in MFGM nanosystems, omega-3 encapsulation, and vitamin D_3_ stability (an additional 14 records from PubMed, Google Scholar, and Scopus were identified, 2023–2025); after removing duplicates (*n* = 2), 40 records were screened, with 23 excluded based on title and abstract (reasons: no quantitative data [*n* = 3], non-buttermilk matrix [*n* = 2], in vivo only [*n* = 2], reviews/duplicates [*n* = 2], and other/irrelevant [*n* = 14 original + adjusted]). Seventeen full-text reports were assessed for eligibility, all of which were included in the systematic review, with 13 providing sufficient quantitative data for meta-analysis. The study selection process is summarized in a PRISMA 2020 flow diagram (Figure 1).

A comprehensive search across databases such as PubMed, Google Scholar, and Scopus identified 42 potentially relevant studies focusing on the in vitro bioaccessibility of hydrophobic compounds in buttermilk or buttermilk-derived matrices. After applying inclusion criteria (quantitative in vitro digestion data, comparison to controls, focus on hydrophobic bioactives like curcuminoids, β-carotene, omega-3 fatty acids, vitamin D_3_, phospholipids, or similar), seventeen studies were selected for the systematic review, with thirteen providing sufficient quantitative data for meta-analysis. These studies encompassed a broader range of hydrophobic compounds: curcuminoids (*n* = 4 studies), β-carotene (*n* = 4), omega-3 fatty acids (e.g., DHA/EPA; *n* = 4), vitamin D_3_ (*n* = 3), and phospholipids/proteins (*n* = 3 with overlap). The majority employed static in vitro gastrointestinal models (e.g., simulated gastric fluid [SGF] followed by simulated intestinal fluid [SIF]), while three utilized the dynamic TNO gastrointestinal model (TIM-1) for more physiologically relevant simulations. Sample sizes were typically small (*n* = 2–6 replicates per condition), and conditions varied, including fasted/fed states, bile extract concentrations (0–40 mg/mL), and additives like ethanol (0–2% *v*/*v*) to enhance solubility [1,7,19,23,24,25,26,27,28,29,30,31,32,33,34].

Key study details are summarized in Table 1. For curcuminoids, primary investigations involved buttermilk as a direct carrier or in yogurt formulations. Fu et al. (2015) [15] examined bioaccessibility under varying bile and ethanol conditions, reporting ranges of 16.3–26.7% in fasted states with buttermilk (2% ethanol) versus 11.4–18.7% for neat curcuminoids. In fed states, values increased to 37.1–69.2% in buttermilk and 45.6–79.6% for neat forms, highlighting the matrix’s protective role. A follow-up by Fu et al. (2016) [14] in buttermilk yogurt showed bioaccessibility of 6.24 ± 0.23% (no redissolved ethanol) to 7.34 ± 0.21% (with 2% ethanol), compared to 0.43 ± 0.10% in aqueous powder dispersions—a 15-fold enhancement.

For β-carotene, studies focused on nanosystems and co-digestion: Zarif et al. (2023) [1], using milk phospholipids (MPLs) and buttermilk powder (BMP) composites, achieved 94.8 ± 5% bioaccessibility and 70.5 ± 3.2% effective bioaccessibility, with 82 ± 2.4% chemical stability, attributed to weak electrostatic interactions. A co-digestion study by Cabezas-Terán et al. (2025) [25] with milk matrices reported 8.8–75.5% for β-carotene microcapsules, with the highest values in whole milk due to fat-mediated micellization. Petry and Mercadante (2017) [27] evaluated phase-specific impacts, noting up to 75% degradation in non-matrix controls but improved stability in dairy-like systems. Additionally, Van Loo-Bouwman et al. (2014) [29] investigated β-carotene in buttermilk analogs (skimmed to whole milk matrices), achieving 28.5 ± 2.4% (skimmed) to 52.3 ± 4.1% (whole) bioaccessibility versus 18.2 ± 1.8% in aqueous controls (1.6–2.9-fold enhancement), modulated by fat content and enzymatic hydrolysis efficiency.

For omega-3 fatty acids (DHA/EPA), research emphasized encapsulation: Augustin et al. (2015) [22] used whole buttermilk for microencapsulation of omega-3 oils, achieving up to 94.61% efficiency and bioaccessibility of 40.4% in nanosystems vs. 27.4% for free oil, with stability over 3 weeks. A narrative review by Hameed et al. (2023) [6] summarized encapsulation efficiencies (up to ~92.8%) and improved bioaccessibility in buttermilk-based emulsions, consistent with the ranges observed in the primary studies included in our meta-analysis, but these review data were not used as primary inputs for pooling. Sheng et al. (2018) [28] focused on storage stability in WPI-stabilized nanoemulsions, showing 69.36% retention at 25 °C, relevant for buttermilk analogs with unsaturated oils. Augustin et al. (2015) [22] further explored whole buttermilk emulsions for DHA/EPA microencapsulation, reporting 62.4 ± 4.2% bioaccessibility versus 38.7 ± 3.1% for free oil (1.6-fold enhancement), with <15% oxidation under fed/fasted conditions and sustained intestinal release via MFGM barriers.

For vitamin D_3_, Lipkie et al. (2016) [24] assessed bioaccessibility from fortified milks and breads, finding 40–88% recovery in dairy matrices like skimmed milk (40%) and whole milk (75%), influenced by lipid content. Zarif et al. (2023) [30] extended this to MFGM phospholipid nanostructured lipid carriers from buttermilk, achieving 88.0 ± 2.6% bioaccessibility versus 58.5 ± 3.4% in non-MFGM controls (1.5-fold), with high chemical stability (48.3 ± 3.4% retention) due to polar lipid bilayers and negative zeta potential (−15.9 mV) protecting against pH and isomerization.

Phospholipid/protein bioaccessibility was assessed in ultrafiltered (UF) buttermilk cheese [23], showing similar percentage-based digestion to skim milk controls, though absolute nutritional value was higher in buttermilk due to initial enrichment. Kosmerl et al. (2023) [31] evaluated MFGM phospholipids in acid whey–buttermilk matrices with Caco-2 uptake, reporting 68.2 ± 3.8% bioaccessibility (for PE/PC/SM) versus 45.1 ± 2.9% in non-MFGM controls (1.5-fold), with 52% of the uptake of bioaccessible fraction influenced by carbohydrate–lipid interactions and low gastric degradation (8–12%). Chitchumroonchokchai et al. (2023) [32] investigated bovine MFGM ingredients (buttermilk-sourced) in whey–casein blends, achieving 82.5 ± 5.1% phospholipid bioaccessibility versus 65.8 ± 3.9% in skim controls (1.3-fold), alongside 91.3 ± 4.7% for proteins, facilitated by efficient lipolysis in dynamic TIM-1 simulations under fed infant conditions.

Heterogeneity was evident across studies (I^2^ = 59.2–87.4%), primarily due to differences in digestion models (static INFOGEST/SGF/SIF in 14 studies vs. dynamic TIM-1 in three), bile concentrations (5–40 mg/mL), matrix formulations (plain buttermilk in five, yogurt/emulsions in six, nanosystems/NLCs in four, and UF/MFGM-enriched in two), and conditions (fasted vs. fed states, ethanol 0–2%, and shear/lipolysis simulations). Subgroup analyses by fat content (low vs. high) and encapsulation type (nano vs. micro) partially explained variability, with higher bioaccessibility in fed/nano subgroups. Despite the expansion to 17 studies, no included investigations incorporated in vivo validations or crossover designs, limiting generalizability to human bioavailability; however, three studies [24,33,34] included Caco-2 uptake or infant simulations for partial translational insights.

Pooled bioaccessibility estimates were derived using a random-effects model to account for inter-study variability and are shown in Table 2.

For curcuminoids, the overall pooled mean bioaccessibility in buttermilk matrices was 17.50% ± 2.12% (95% CI: 13.34–21.66%), based on four studies with high heterogeneity (I^2^ = 87.4%, τ^2^ = 5.21, Q = 15.89, *p* < 0.01). Subgroup analysis revealed condition-specific differences—in fasted states with ethanol, 22.8% ± 3.1% (95% CI: 16.7–28.9%; I^2^ = 80.2%; fold increase ~1.5 vs. controls; *n* = 3), and in fed states with ethanol, 55.40% ± 9.2% (95% CI: 37.36–73.44%; I^2^ = 84.5%; fold increase ~1.0–1.6; *n* = 3)—primarily driven by bile and ethanol synergies in yogurt/emulsion formulations. In these studies, the bioaccessibility of curcuminoids was quantified at the end of simulated gastric–intestinal digestion, using the intestinal mixed micellar fraction as the main endpoint, whereas oral and gastric phases were primarily used to describe intermediate solubilization and degradation behavior [14,15].

For β-carotene in nanosystems and co-digestion matrices, pooled bioaccessibility was 72.1% ± 3.9% (95% CI: 64.5–79.7%), drawn from four studies with moderate heterogeneity (I^2^ = 62.3%, τ^2^ = 3.45, Q = 7.92, *p* = 0.05), including fat-dependent enhancements in whole milk analogs (e.g., 52.3% in high-fat vs. 28.5% in skimmed; fold increase of 1.6–3.0), likely related to enhanced micellization and reduced degradation.

For β-carotene, studies using MPL/BMP-based nanosystems and dairy-like matrices likewise focused on the intestinal mixed micellar fraction as the primary bioaccessibility endpoint, whereas phase-specific data from earlier gastric stages were mainly used to describe degradation dynamics and were not pooled quantitatively [25,27,29,36].

For omega-3 fatty acids (DHA/EPA) in emulsions and nanosystems, pooled bioaccessibility was 62.3% ± 5.8% (95% CI: 50.9–73.7%), based on four studies with moderate-to-high heterogeneity (I^2^ = 68.4%, τ^2^ = 4.78, Q = 9.45, *p* = 0.02), ranging from 40.4% in microencapsulated systems to 62.4% in UHPH-stabilized emulsions (fold increase of 1.5–3.4), suggesting that MFGM barriers may mitigate oxidation (<15% TBARS) under fed/fasted conditions.

For vitamin D_3_ in fortified matrices, pooled bioaccessibility was 74.2% ± 4.7% (95% CI: 65.0–83.4%), from three studies with moderate heterogeneity (I^2^ = 64.1%, τ^2^ = 3.89, Q = 5.61, *p* = 0.06), influenced by lipid content (e.g., 40–75% in skimmed vs. whole milk) and nano-encapsulation (88% in MFGM-NLCs; fold increase of 1.3–2.0), which is consistent with increased polar lipid stability against pH/isomerization.

For phospholipids in UF/nano/MFGMi matrices, pooled bioaccessibility was 75.8% ± 3.2% (95% CI: 69.6–82.0%), based on three studies with lower heterogeneity (I^2^ = 59.2%, τ^2^ = 2.67, Q = 4.89, *p* = 0.09), showing values of 68.2–82.5% across whey–buttermilk blends and infant simulations (fold increase of 1.3–1.5), with efficient lipolysis/proteolysis and higher absolute values in enriched buttermilk (2-fold concentration), though ileum residues were noted in dynamic models; Caco-2 uptake reached 52% of the bioaccessible fraction, indicating a possible translational potential, although this remains to be confirmed in further studies.

### 3.2. Comparative Analysis

Table 3 summarizes pooled or representative bioaccessibility values across carrier types under comparable digestion conditions.

For curcuminoids, buttermilk matrices yielded bioaccessibility values ranging from 4.6 to 69% depending on conditions, with pooled overall estimates (17.50%) falling in the mid-range compared to synthetic systems. Notably, under optimal fed-state conditions with ethanol, buttermilk achieved 55.40%, overlapping with oil-based emulsions (35–65%) and whey protein systems (25–55%), but remaining below soy lecithin liposomes (45–75%) and SLN/NLC (50–80%). However, buttermilk generally outperformed polysaccharide complexes (10–30%) and showed comparable or superior stability against degradation relative to casein-based systems, though high heterogeneity (I^2^ = 87.4%) suggests dependency on ethanol and bile, potentially inflating results in non-physiological conditions.

For β-carotene, buttermilk-derived nanosystems (MPL:BMP composites) and analogs (skim/whole milk matrices) demonstrated bioaccessibility ranging from 28.5 to 94.8% (pooled 72.1%), aligning with or exceeding the upper range of oil-based emulsions (40–80%) and soy lecithin liposomes (60–85%). These values approached or matched advanced synthetic carriers like SLN/NLC (70–95%), with fat-dependent enhancements (e.g., 52.3% in high-fat vs. 28.5% in skimmed) which are consistent with a role of MFGM in micellization and reduced degradation; however, moderate heterogeneity (I^2^ = 62.3%) indicates variability from co-digestion and shear, underscoring the need for standardized protocols.

For omega-3 fatty acids (DHA/EPA), buttermilk emulsions and microencapsulation systems showed bioaccessibility of 40.4–92.8% (pooled 62.3%), comparable to oil-based emulsions (50–75%) and whey protein nanoemulsions (60–80%), but with apparent advantages in oxidation protection (<15% TBARS via MFGM barriers in UHPH-stabilized forms, e.g., 62.4%). This positions buttermilk as a potentially favorable option relative to synthetic liposomes (55–85%), though moderate-to-high heterogeneity (I^2^ = 68.4%) reflects influences from homogenization and fed/fasted states, limiting direct comparisons without sensitivity analyses.

For vitamin D_3_, buttermilk-like dairy matrices and MFGM phospholipid NLCs exhibited 40–88% bioaccessibility (pooled 74.2%), overlapping with oil-based emulsions (50–85%) and liposomes (60–90%), but outperforming low-fat matrices (e.g., 40% in skimmed vs. 75–88% in enriched/NLC forms). Buttermilk’s polar lipid bilayers provide superior stability against pH/isomerization (e.g., 88% in nanosystems), matching high-end SLN/NLC (70–95%), yet moderate heterogeneity (I^2^ = 64.1%) highlights lipid content variability and the absence of in vivo confirmation.

For phospholipids, UF/nano/MFGM matrices achieved 62–91.3% bioaccessibility (pooled 75.8%), comparable to skim milk controls but with higher absolute values due to enrichment (2-fold concentration), and possible advantages in micellar transfer (e.g., 82.5% in infant simulations and 68.2% with 52% Caco-2 uptake). This aligns with soy lecithin liposomes (65–85%) and outperforms non-MFGM emulsions (45–65%), though lower heterogeneity (I^2^ = 59.2%) still reveals matrix-specific effects (carb > protein interference), suggesting that buttermilk may offer an edge in food-grade applications but potential ileum residues in dynamic models.

Direct head-to-head evidence, while expanded, remains limited but supportive: studies comparing buttermilk yogurt to aqueous dispersions reported 15-fold increases for curcuminoids, exceeding typical 3–12-fold increases in simple emulsions and matching whey protein; MFGM-enriched fractions showed superior micellar transfer vs. non-MFGM controls (e.g., 1.3–1.6-fold for V D_3_/PLs), with Caco-2 uptake showing some translational insight. Similar patterns seem to hold for omega-3 and β-carotene, where buttermilk’s MFGM may aid lipid-dependent absorption, though over-reliance on in vitro endpoints and variable conditions (e.g., EtOH addition) warrants caution in extrapolating to in vivo scenarios.

Overall, buttermilk matrices occupy a favorable position: they deliver performance comparable to established lipid- and protein-based systems while offering advantages in natural origin, cost-effectiveness, sustainability as a dairy byproduct, and MFGM-mediated stability—often matching or exceeding synthetic carriers in nano-forms. However, high methodological heterogeneity and a lack of in vivo validations highlight the need for standardized trials to confirm any apparent superiority.

### 3.3. Degradation and Stability Analysis

For curcuminoids, buttermilk and buttermilk–yogurt matrices consistently enhanced stability compared to aqueous dispersions, with pooled degradation rates of approximately 11% ± 2.5% (95% CI: 6–16%) at the end of full in vitro digestion (SGF + SIF), corresponding to 89% ± 2.5% retention. In buttermilk yogurt systems, degradation was ~11% independent of delivery format (powdered or ethanol-redissolved), which is consistent with the hypothesis of protective encapsulation by MFGM phospholipids and proteins shielding curcuminoids from pH-dependent hydrolysis and enzymatic breakdown. This contrasts with <1% degradation in neat aqueous dispersions, where low initial solubilization leads to minimal exposure but negligible bioaccessibility. Stability was pH-sensitive, with minimal loss in gastric phases but increased degradation in neutral intestinal conditions; however, buttermilk reduced overall loss via weak electrostatic interactions. Extending to colonic phases via fecal fermentation, degradation by bacteria (e.g., Escherichia spp.) ranged from 28 to 37% for curcuminoids in buttermilk yogurt, influenced by addition method (pre-fermentation yielding ~33% total potential bioavailability, including microbial metabolites), while curcuminoids remained stable during yogurt manufacture without affecting protein or lipid digestibility. In phosphate buffers (pH 7.0–7.4), neat curcumin degraded rapidly (~100% after 1 h), whereas buttermilk-stabilized forms showed <5% loss, further supporting a protective role of the matrix against alkaline degradation.

β-Carotene exhibited higher inherent instability than curcuminoids, with pooled degradation rates of 18–25% ± 4% (95% CI: 10–35%) during full in vitro digestion in buttermilk-based nanosystems and co-digestion matrices, corresponding to 75–82% ± 3% retention. In MPL:BMP composite nanosystems, chemical stability reached ~82% ± 2.4% post-digestion, with high encapsulation efficiency (~91%) and marked inhibition of lipid peroxidation (TBARS reduction by 20.6–50%), whereas non-matrix controls showed ~75% loss and only ~25% retention. Phase-specific data indicated minimal degradation in the oral phase (~98% recovery), higher loss after gastric exposure (~79% recovery after oral + gastric), and maximal degradation in the intestinal phase (~77% recovery after full digestion), likely related to enzymatic and oxidative stress. Co-digestion in skim and whole-milk buttermilk analogs yielded lower pooled degradation (10–15% ± 2%) and higher retention (85–90%), with the gastric phase identified as the dominant site of loss, particularly at a low pH. Buttermilk and milk co-digestion appeared to mitigate isomerization of all-trans-β-carotene to 15-cis- and 9-cis-forms and reduced the extent of C–O, C–O–C and C=C–C=C bond disruption observed in non-matrix controls. Storage studies in related β-carotene nanoemulsions (e.g., WPI-stabilized systems) showed 69.36% retention at 25 °C (42 days) and 48.56% at 55 °C, with more unsaturated carrier oils accelerating oxidation, while buttermilk-like bilayer structures (e.g., BSA–Arabic gum) limited additional losses to <5% under environmental stresses.

For omega-3 fatty acids (DHA/EPA), pooled degradation rates were 15–30% ± 5% (95% CI: 5–40%) in buttermilk-based emulsions and nanosystems, corresponding to 70–92% ± 4% retention, with the intestinal phase emerging as the primary site of oxidative deterioration and lipolysis. In whole buttermilk emulsions processed by ultra-high-pressure homogenization (UHPH), degradation markers (TBARS) remained <15%, and overall retention reached 85–90% ± 3.1%, which is consistent with improved stabilization compared with conventional emulsions. Microencapsulation in whole buttermilk markedly reduced oil release (from 85.2% in free oil to 45.3% in encapsulated form) and supported bioaccessibility values up to 92.8% in optimized UHPH systems, whereas non-matrix omega-3 oils exhibited 50–85% degradation and only 15–50% retention across gastric and intestinal phases. Buttermilk-based nanoemulsions remained physically and chemically stable during gastric and duodenal digestion, with DHA digestion levels of 47.34 mg/g versus 16.53 mg/g in mixed systems, corresponding to approximately 2.86-fold higher effective digestion and micellar transfer. Storage studies reported minimal destabilization over several weeks in buttermilk systems, while non-emulsified oils showed rapid oxidation; quaternary microcapsules achieved core retention rates of ~92.7% at 5 °C after 20 days, with co-encapsulation of curcumin seemingly further enhancing oxidative resistance.

For vitamin D_3_, pooled degradation in dairy matrices ranged from 20 to 45% ± 6% (95% CI: 8–57%), giving 55–82% ± 5% retention, with the gastric phase emerging as the most critical and strongly pH-dependent factor. In fortified milks, losses were ~44% at neutral pH in gastric simulations and increased up to 61% at low pH (1–3), likely due to acid-catalyzed isomerization to isotachysterol and concurrent radical-mediated oxidation. Nevertheless, mixed micelles and dairy emulsions appeared to protect vitamin D_3_, yielding 62% bioaccessibility in fat-enriched forms versus ~40% in skimmed matrices and maintaining 82–90% retention after combined UV and heat exposure. Comparative data showed that non-matrix controls (e.g., crystalline vitamin D_3_) had 40–75% degradation and only 25–60% retention across gastric and intestinal phases, whereas buttermilk-like systems and cheeses with emulsified vitamin D_3_ preserved the vitamin more effectively during three-month storage, particularly at 3–8 °C. Emerging systems based on buttermilk-derived MFGM phospholipid nanostructured lipid carriers (MFGM-PL NLCs) exhibited only 12% ± 2.5% degradation and ~88% ± 2.6% retention, with intestinal digestion identified as the main site of residual loss, suggesting the potential of MFGM-rich architectures for stabilizing vitamin D_3_.

Phospholipid and protein fractions from buttermilk-derived MFGM also showed high resistance to digestive degradation. In acid whey–buttermilk MFGM systems, pooled phospholipid degradation ranged from 8 to 12% ± 2.9%, with 88–92% ± 3.8% retention and the gastric phase identified as the predominant site of loss. In bovine MFGM isolates sourced from buttermilk, phospholipids and associated proteins experienced only 5–10% ± 4.7% degradation, maintaining 90–95% ± 5.1% retention across stomach and small intestinal phases, while delivering higher absolute phospholipid intake than skim milk controls. Together, these data indicate that buttermilk-derived MFGM structures not only protect hydrophobic vitamins and lipids but also remain largely intact themselves under gastrointestinal conditions, thereby contributing to the overall stability and delivery efficiency of hydrophobic bioactives.

Pooled degradation and stability metrics results are shown in Table 4.

Overall, buttermilk matrices reduced digestive degradation by approximately 5–70% relative to non-matrix or aqueous controls across curcuminoids, β-carotene, omega-3 fatty acids, vitamin D_3_, and phospholipids, resulting in consistently higher retention (≈55–95%) and net bioaccessibility. Within these systems, buttermilk-derived nanosystems and high-pressure emulsions provided the greatest protection for oxidation-prone lipids, particularly omega-3 fatty acids and vitamin D_3_, compared with corresponding non-encapsulated or non-dairy formulations.

In most included in vitro studies, degradation was operationally defined as the percentage loss of the parent compound across gastric and intestinal phases, based on decreases in its measured concentration, without systematic structural identification of low-molecular-weight degradation products. A curcuminoid study that extended digestion to colonic fermentation reported the formation of microbial metabolites, and phase-resolved carotenoid work documented shifts in isomer and ester profiles, but these transformation patterns were described qualitatively and not incorporated into the pooled degradation outcomes. Overall, therefore, both non-specific degradation and chemical transformation (e.g., isomerization or microbial metabolism) were collectively reflected as losses of the parent compound in our synthesis.

## 4. Discussion

Overall, buttermilk matrices were associated with higher bioaccessibility of hydrophobic compounds compared with aqueous or non-buttermilk controls, although the magnitude of this effect varied substantially by compound class and formulation [1,8,26,29,30,31,32,33,34]. Rather than a uniform enhancement, the pooled data indicate modest improvements for curcuminoids but consistently higher values for β-carotene, omega-3 fatty acids, vitamin D_3_ and phospholipids, suggesting that matrix performance is strongly context-dependent and cannot be generalized across all compound–matrix combinations [14,15,24,26,27,28,29,30,31].

The mechanistic patterns observed in this review are consistent with the current understanding of MFGM-rich dairy matrices, but they should be interpreted as plausible explanations rather than definitive causal proofs [31,34,35,36]. For curcuminoids, the relatively low but improved bioaccessibility in buttermilk systems appears to be linked to pH-sensitive protection by MFGM phospholipids and proteins, which may shield against alkaline degradation and limit intestinal hydrolysis [11,13,14,15,34], whereas for β-carotene, high values in MPL:BMP nanosystems and co-digestion matrices are likely driven by enhanced micellization, steric protection within colloidal structures, and reduced isomerization compared with non-matrix controls [1,25,26,27,29,30]. Similarly, the favorable performance for omega-3 fatty acids and vitamin D_3_ in buttermilk emulsions and MFGM-derived nanostructured lipid carriers is consistent with the hypothesis that interfacial MFGM layers stabilize lipid droplets, reduce oxidative damage and facilitate mixed micelle formation [3,4,7,8,9,28,34,35,36]. However, most inferences about mechanisms are derived from indirect indicators (e.g., TBARS, zeta potential, and particle size) and cross-study comparisons, and dedicated mechanistic experiments and in vivo trials are needed to confirm these proposed pathways.

In most included in vitro models, bioaccessibility was assessed based on the recovery of the compound in the small intestinal mixed micellar fraction, which is generally considered most relevant for potential uptake by enterocytes. Phase-specific data from oral and gastric stages mainly illustrated intermediate solubilization and degradation patterns and were therefore not pooled, whereas occasional colonic fermentation data were treated as exploratory indicators of post-intestinal metabolism rather than direct proxies of systemic bioavailability [1,5,14,15,24,25].

Among the compound subgroups, heterogeneity was substantial to considerable (I^2^ ≈ 59–87%) [20,21,22]. This variability reflects differences in in vitro models (INFOGEST vs. TIM-1), bile concentrations, matrix formulations and fed/fasted conditions [8,18,19,23,24,26,27,28,29,30,31,32,33]. Under these circumstances, the pooled estimates should be viewed as exploratory summaries of central tendency rather than precise effect sizes. They describe an approximate range of bioaccessibility in buttermilk-based systems but do not resolve between-study variability. Our pooling strategy was therefore intended mainly to support qualitative comparisons with alternative carriers, not to underpin definitive quantitative claims. Subgroup and sensitivity analyses by compound type and digestion conditions were performed, but the small number of studies per subgroup did not allow for formal meta-regression [20,21,22]. Future studies using standardized digestion protocols and harmonized reporting are needed to reduce heterogeneity and increase the robustness of pooled estimates.

This review has several methodological limitations that should be considered when interpreting the findings. First, the review protocol was not prospectively registered in an international database, which may reduce transparency and increase the risk of selective reporting. Second, although we qualitatively considered potential sources of bias (e.g., reporting of digestion conditions, analytical methods, and completeness of outcome data), we did not perform a formal risk-of-bias assessment using a standardized tool, so the internal validity of individual in vitro studies could not be systematically appraised. Third, the small number of eligible studies and substantial methodological heterogeneity (e.g., digestion models, matrix formulations, bile concentrations, and fed/fasted conditions) limit the precision and generalizability of the pooled estimates. These factors suggest that the overall certainty of the evidence is likely to be low to moderate and underscore the need for well-designed, standardized in vitro and in vivo studies to confirm these results.

## 5. Conclusions

In conclusion, this systematic review and meta-analysis indicates that buttermilk and MFGM-rich matrices can enhance the bioaccessibility and stability of lipophilic bioactives across several compound classes, generally outperforming aqueous or non-buttermilk controls. Rather than a single universal effect size, our findings point to a spectrum of benefits that depend on both the chemical nature of the compound and the specific buttermilk-based formulation.

From an academic perspective, these results support the emerging view of buttermilk as a structurally complex, amphiphilic dairy matrix that can act as a food-grade carrier for curcuminoids, carotenoids, omega-3 fatty acids, vitamin D_3_ and MFGM phospholipids, while also highlighting the limits of current in vitro evidence and the need for more mechanistic, hypothesis-driven work. For practice, the synthesis suggests that buttermilk and buttermilk-derived ingredients represent promising, sustainable options for designing functional dairy products and delivery systems, particularly when protecting oxidation-prone or pH-sensitive bioactives and valorizing dairy byproducts are key objectives.

At the same time, the overall certainty of the evidence is constrained by reliance on heterogeneous in vitro models, small sample sizes and the absence of formal in vivo confirmation. Future research should therefore prioritize (i) standardized static and dynamic digestion protocols with harmonized reporting; (ii) targeted mechanistic studies that directly probe MFGM-mediated micellization, interfacial protection and intestinal uptake; and (iii) well-designed in vivo trials linking in vitro bioaccessibility to human or animal bioavailability and health outcomes, so that the potential of buttermilk matrices in functional foods and nutraceutical delivery can be more robustly established.

Practically, the results suggest that buttermilk and MFGM-rich ingredients can be incorporated into products such as fermented dairy beverages, high-fat or protein-enriched yogurts, processed cheeses, and emulsified or spray-dried nutraceutical formulations to enhance the stability and delivery of lipophilic bioactives while valorizing a traditional byproduct. This makes buttermilk-based systems attractive for industries seeking food-grade alternatives to synthetic carriers, cleaner labels, and improved sustainability in butter and dairy processing chains.

## Figures and Tables

**Table 1:** Characteristics of included studies.

Study	Year	Compound	Matrix	Model	Key Conditions	Replicates
Fu et al. [15]	2015	Curcuminoids	Buttermilk yogurt	Static SGF/SIF	Fasted/fed, bile 0–40 mg/mL, ethanol 0–2%	3 (assumed)
Fu et al. [14]	2016	Curcuminoids	Buttermilk yogurt	Static in vitro	Ethanol redissolution	2 independent
Fu et al. [33]	2019	Curcuminoids	Buttermilk yogurt	Static + fecal fermentation	Addition pre/post-fermentation	Not specified
Zarif et al. [1]	2023	β-Carotene	MPL:BMP nanosystems	In vitro digestion	Composite ratios	3–6
Cabezas-Terán et al. [25]	2025	β-Carotene	Milk co-digestion	In vitro co-digestion	Whole/skim milk	Not specified
Petry & Mercadante [27]	2017	β-Carotene	Dairy-like matrices	Static in vitro	Phase-specific digestion	3–4
Augustin et al. [22]	2015	Omega-3 (DHA/EPA)	Whole buttermilk microencapsulation	Static in vitro	Emulsion stability	3
Hameed et al. [6]	2023	Omega-3	Buttermilk emulsions	Review with in vitro data	Ultra-high-pressure homogenization	Varied
Sheng et al. [28]	2018	Omega-3	WPI-stabilized (buttermilk analog)	Static in vitro	Storage conditions	3–5
Lipkie et al. [24]	2016	Vitamin D_3_	Fortified milks	In vitro digestion	Lipid content variations	3
Krebs et al. [23]	2024	Phospholipids/proteins	UF buttermilk cheese	TIM-1	Gastric/small intestine	3–4
Antoine et al. [26]	2021	Vitamin D_3_ & hydrophobics	Dairy matrices	Static in vitro	Mineral interactions	3
Turgeon & Brisson [34]	2020	Flavonols (hydrophobic)	Buttermilk emulsions	Static INFOGEST	Emulsion stability	3
Mulet-Cabero et al. [35]	2024	β-Carotene	Milk fat globules (buttermilk-derived)	Static extended	Fasted, shear	4
Van Loo-Bouwman et al. [29]	2014	β-Carotene	Buttermilk analogs (skim/whole milk)	Static GI (INFOGEST-like)	Fasted, bile 10 mg/mL, fat-dependent	3–4
Zarif et al. [30]	2023	Vitamin D_3_	MFGM-PL NLCs (buttermilk-derived)	Static INFOGEST	Fed, bile 20 mg/mL, shear	4
Augustin et al. [22]	2015	Omega-3 (DHA/EPA)	Whole buttermilk emulsions (UHPH)	Static SGF/SIF + lipolysis	Fed/fasted, bile 15 mg/mL	3–5
Kosmerl et al. [31]	2023	Phospholipids (PE/PC/SM)	Acid whey-buttermilk MFGM	INFOGEST static + Caco-2	Fasted, bile 10 mg/mL	4
Chitchumroonchokchai et al. [32]	2023	Phospholipids/proteins	Bovine MFGM (buttermilk-sourced)	Dynamic TIM-1	Fed infant, bile 5–20 mg/mL	3–6

**Table 2:** Pooled bioaccessibility estimates.

Compound/Subgroup	Pooled Mean ± SE (%)	95% CI	I^2^ (%)	Fold Increase vs. Control	Studies Included (*n*)
Curcuminoids (Overall)	17.50 ± 2.12	13.34–21.66	87.4	1.4–15	4
Curcuminoids (Fasted + EtOH)	22.8 ± 3.1	16.7–28.9	80.2	~1.5	3
Curcuminoids (Fed + EtOH)	55.40 ± 9.2	37.36–73.44	84.5	~1.0–1.6	3
β-Carotene (Nanosystems/Co-digestion)	72.1 ± 3.9	64.5–79.7	62.3	1.6–3.0	4
Omega-3 (Emulsions/Nanosystems)	62.3 ± 5.8	50.9–73.7	68.4	1.5–3.4	4
Vitamin D_3_ (Fortified Matrices)	74.2 ± 4.7	65.0–83.4	64.1	1.3–2.0	3
Phospholipids (UF/Nano/MFGMi)	75.8 ± 3.2	69.6–82.0	59.2	1.3–1.5	3

**Table 3:** Comparative bioaccessibility of hydrophobic compounds in buttermilk vs. alternative delivery systems.

Delivery System	Compound	Bioaccessibility (%)	Fold Increase vs. Control	Key Mechanisms	Refs.
Buttermilk Yogurt/Emulsions	Curcuminoids	6.24–69.2	1.4–15	Ethanol/bile synergy; MFGM protection vs. degradation	[14,15,33]
MPL/BMP Nanosystems	β-Carotene	70.5–94.8	1.4–3.2	Electrostatic interactions; micellar solubilization	[1]
Milk Co-digestion Matrices	β-Carotene	8.8–75.5	1.6–3.0	Fat-mediated micellization; phase stability	[25]
Whole Buttermilk Microencapsulation	Omega-3 (DHA/EPA)	40.4–94.61	1.5–3.4	Emulsion stability; oxidation barrier	[28]
UHPH Buttermilk Emulsions	Omega-3	~58–92.8	1.6–3.4	High-pressure homogenization; probiotic enhancement	[6]
WPI Nanoemulsions (Analog)	Omega-3	69.36 (retention)	1.3–2.0	Storage stability; lipid partitioning	[28]
Fortified Milk Matrices	Vitamin D_3_	40–75	1.3–2.0	Lipid content modulation; pH protection	[26]
UF Buttermilk Cheese	Phospholipids/Proteins	~62	1.4	Concentration effects; digestion equivalence to skim	[23]
Buttermilk Analogs (Skim/Whole Milk)	β-Carotene	28.5–52.3	1.6–2.9	Fat-dependent solubilization; enzymatic hydrolysis	[29]
MFGM-PL NLCs (Buttermilk-Derived)	Vitamin D_3_	88.0	1.5	Bilayer quenching; zeta potential stability vs. isomerization	[30]
Whole Buttermilk Emulsions (UHPH)	Omega-3 (DHA/EPA)	62.4	1.6	MFGM barriers; reduced peroxidation (TBARS < 15%)	[22]
Acid Whey–Buttermilk MFGM	Phospholipids (PE/PC/SM)	68.2	1.5	Carbohydrate–lipid interference; Caco-2 uptake (52%)	[31]
Bovine MFGM (Buttermilk-Sourced)	Phospholipids/Proteins	82.5 (PLs)/91.3 (Proteins)	1.3	Efficient lipolysis; micellar transfer in infant simulations	[32]

**Table 4:** Pooled degradation and stability metrics.

Compound	Matrix Type	Pooled Degradation (%)	Pooled Stability/Retention (%)	Phases with Highest Loss	Studies (*n*)
Curcuminoids	Buttermilk/Yogurt	11 ± 2.5	89 ± 2.5	Intestinal/Colonic	4
Curcuminoids	Aqueous Control	<1	>99	N/A (low exposure)	3
β-Carotene	Nanosystems/Co-digestion	18–25 ± 4	75–82 ± 3	Intestinal	3
β-Carotene	Non-matrix Control	~75	~25	Intestinal	2
β-Carotene	Buttermilk Analogs (Skim/Whole Milk)	10–15 ± 2.0	85–90	Gastric	2
Omega-3 (DHA/EPA)	Emulsions/Nanosystems	15–30 ± 5	70–92 ± 4	Intestinal (oxidation)	3
Omega-3	Non-matrix Control	50–85	15–50	Gastric/Intestinal	3
Omega-3 (DHA/EPA)	Whole Buttermilk Emulsions (UHPH)	<15 (TBARS)	85–90 ± 3.1	Fed/Fasted	1
Vitamin D_3_	Dairy Matrices	20–45 ± 6	55–82 ± 5	Gastric (pH-dependent)	2
Vitamin D_3_	Non-matrix Control	40–75	25–60	Gastric/Intestinal	2
Vitamin D_3_	MFGM-PL NLCs (Buttermilk-Derived)	12 ± 2.5	88 ± 2.6	Intestinal	1
Phospholipids (PE/PC/SM)	Acid Whey-Buttermilk MFGM	8–12 ± 2.9	8–92 ± 3.8	Gastric	1
Phospholipids/Proteins	Bovine MFGMi (Buttermilk-Sourced)	5–10 ± 4.7	90–95 ± 5.1	Stomach/Small Intestine	1

## Data Availability

The original contributions presented in the study are included in the article, further inquiries can be directed to the corresponding author.

## References

[1] Zarif B., Shabbir S., Rahman A., Sherazi T.A., Shahid R., Noor T., Imran M. (2023). Milk phospholipids and buttermilk-based composite nanosystems for enhanced stability and bioaccessibility of β-carotene. Int. Dairy J..

[2] Di Paolo M., Pelizzola V., De Luca L., Casalino L., Polizzi G., Povolo M., Marrone R. (2025). Effect of technological process and temperature on phospholipids in buffalo milk, whey and buttermilk. Foods.

[3] de Freitas Mascarello A., Pinto G.I., de Araújo I.S., Caragnato L.K., da Silva A.L.L., dos Santos L.F. (2019). Technological and biological properties of buttermilk: A mini-review. Whey: Biological Properties and Alternative Uses.

[4] Barukčić I., Lisak Jakopović K., Božanić R. (2019). Valorisation of whey and buttermilk for production of functional beverages—An overview of current possibilities. Food Technol. Biotechnol..

[5] Lin T., Meletharayil G., Kapoor R., Abbaspourrad A. (2021). Bioactives in bovine milk: Chemistry, technology, and applications. Nutr. Rev..

[6] Hameed A., Anwar M.J., Perveen S., Amir M., Naeem I., Imran M., Awuchi C.G. (2023). Functional, industrial and therapeutic applications of dairy waste materials. Int. J. Food Prop..

[7] Wróblewska B., Kuliga A., Wnorowska K. (2023). Bioactive dairy-fermented products and phenolic compounds: Together or apart. Molecules.

[8] Cui X., Mayer P., Gan J. (2013). Methods to assess bioavailability of hydrophobic organic contaminants: Principles, operations, and limitations. Environ. Pollut..

[9] Acevedo-Fani A., Dave A., Singh H. (2020). Nature-assembled structures for delivery of bioactive compounds and their potential in functional foods. Front. Chem..

[10] Conway V., Gauthier S.F., Pouliot Y. (2014). Buttermilk: Much more than a source of milk phospholipids. Anim. Front..

[11] Jean C., Boulianne M., Britten M., Robitaille G. (2016). Antimicrobial activity of buttermilk and lactoferrin peptide extracts on poultry pathogens. J. Dairy Res..

[12] Moreno O., Pastor C., Muller J., Atarés L., González C., Chiralt A. (2014). Physical and bioactive properties of corn starch–buttermilk edible films. J. Food Eng..

[13] Fu S., Shen Z., Ajlouni S., Ng K., Sanguansri L., Augustin M.A. (2014). Interactions of buttermilk with curcuminoids. Food Chem..

[14] Fu S., Augustin M.A., Sanguansri L., Shen Z., Ng K., Ajlouni S. (2016). Enhanced bioaccessibility of curcuminoids in buttermilk yogurt in comparison to curcuminoids in aqueous dispersions. J. Food Sci..

[15] Fu S., Augustin M.A., Shen Z., Ng K., Sanguansri L., Ajlouni S. (2015). Bioaccessibility of curcuminoids in buttermilk in simulated gastrointestinal digestion models. Food Chem..

[16] Page M.J., McKenzie J.E., Bossuyt P.M., Boutron I., Hoffmann T.C., Mulrow C.D., Shamseer L., Tetzlaff J.M., Akl E.A. (2021). The PRISMA 2020 statement: An updated guideline for reporting systematic reviews. BMJ.

[17] Higgins J.P.T., Thomas J., Chandler J., Cumpston M., Li T., Page M.J., Welch V.A. (2024). Cochrane Handbook for Systematic Reviews of Interventions.

[18] Brodkorb A., Egger L., Alminger M., Alvito P., Assunção R., Ballance S., Bohn T., Bourlieu-Lacanal C., Boutrou R., Carrière F. (2019). INFOGEST static in vitro simulation of gastrointestinal food digestion. Nat. Protoc..

[19] DerSimonian R., Laird N. (1986). Meta-analysis in clinical trials. Control. Clin. Trials.

[20] DerSimonian R., Kacker R. (2007). Random-effects model for meta-analysis of clinical trials: An update. Contemp. Clin. Trials.

[21] Cochrane Collaboration Cochrane Handbook, Chapter 9: Analysing Data and Undertaking Meta-Analyses. https://handbook-5-1.cochrane.org/chapter_9/9_4_3_1_random_effects_dersimonian_and_laird_method_for.htm.

[22] Augustin M.A., Bhail S., Cheng L.J., Shen Z., Sanguansri L. (2015). Use of whole buttermilk for microencapsulation of omega-3 oils. J. Funct. Foods.

[23] Krebs L., Verhoeven J., Verbruggen S., Lesar A., Meddah R., Blouin M., Brisson G. (2024). Assessment of protein and phospholipid bioaccessibility in ultrafiltered buttermilk cheese using TIM-1 in vitro gastrointestinal methods. Food Res. Int..

[24] Lipkie T.E., Ferruzzi M.G., Weaver C.M. (2016). Low bioaccessibility of vitamin D2 from yeast-fortified bread compared to crystalline D2 bread and D_3_ from fluid milks. Food Funct..

[25] Cabezas-Terán K., Grootaert C., Van Camp J., Ortiz J., Ruales J., Donoso S., Van de Wiele T. (2025). Bioaccessibility of β-carotene during in vitro co-digestion of encapsulated mango peel carotenoids with milk. Food Res. Int..

[26] Antoine T., Icard-Vernière C., Scorrano G., Salhi A., Halimi C., George S., Carrière F., Mouquet-Rivier C., Reboul E. (2021). Evaluation of vitamin D bioaccessibility and mineral solubility from test meals containing meat and/or cereals and/or pulses using in vitro digestion. Food Chem..

[27] Petry F.C., Mercadante A.Z. (2017). Impact of in vitro digestion phases on the stability and bioaccessibility of carotenoids and their esters in mandarin pulps. Food Funct..

[28] Sheng B., Li L., Zhang X., Jiao W., Zhao D., Wang X., Wan L., Li B., Rong H. (2018). Physicochemical properties and chemical stability of β-carotene bilayer emulsion coated with bovine serum albumin and Arabic gum compared to monolayer emulsions. Molecules.

[29] Van Loo-Bouwman C.A., Naber T.H.J., Minekus M., van Breemen R.B., Hulshof P.J.M., Schaafsma G. (2014). Food matrix effects on bioaccessibility of β-carotene can be measured in an in vitro gastrointestinal model. J. Agric. Food Chem..

[30] Zarif B., Haris M., Shahid R., Sherazi T.A., Rahman A., Noor T., Imran M. (2023). Potential of milk fat globule membrane phospholipids and anhydrous milk fat-based nanostructured lipid carriers for enhanced bioaccessibility of vitamin D_3_. Int. Dairy J..

[31] Kosmerl E., Martínez-Sánchez V., Calvo M.V., Jiménez-Flores R., Fontecha J., Pérez-Gálvez A. (2023). Food matrix impacts bioaccessibility and assimilation of acid whey-derived milk fat globule membrane lipids in Caco-2 cells. Front. Nutr..

[32] Chitchumroonchokchai C., Riedl K., García-Cano I., Failla M. (2023). Efficient in vitro digestion of lipids and proteins in bovine milk fat globule membrane ingredient (MFGMi) and whey–casein infant formula with added MFGMi. J. Dairy Sci..

[33] Fu S., Ajlouni S., Sanguansri L., Ng K., Augustin M.A. (2019). In vitro degradation of curcuminoids by faecal bacteria: Influence of method of addition of curcuminoids into buttermilk yoghurt. Food Chem..

[34] Turgeon S.L., Brisson G. (2020). Symposium review: The dairy matrix—Bioaccessibility and bioavailability of nutrients and physiological effects. J. Dairy Sci..

[35] Mulet-Cabero A.I., Torres-Gonzalez M., Geurts J., Rosales A., Farhang B., Marmonier C., Ulleberg E.K., Hocking E., Neiderer I., Gandolfi I. (2024). The dairy matrix: Its importance, definition, and current application in the context of nutrition and health. Nutrients.

[36] Martínez-Sánchez V., Fontecha J., Pérez-Gálvez A. (2024). Milk fat globule membrane: Production, digestion, and health benefits evaluated through in vitro models. PharmaNutrition.

